# EMX2OS serves as a biomarker of neonatal sepsis and participates acute lung injury through enhancing ferroptosis

**DOI:** 10.3389/fped.2025.1654832

**Published:** 2025-09-09

**Authors:** Xuexiang Li, Zhiqiang Liu, Guilian Shan, Lili Shi, Zhihua Liu

**Affiliations:** Department of Neonatology, Shengli Oilfield Central Hospital, Dongying, Shandong, China

**Keywords:** neonatal sepsis, acute lung injury, ferroptosis, viability, EMX2OS, miR-654-3p/AKT3

## Abstract

**Background:**

Neonatal Sepsis (NS) is an important cause of neonatal death, often accompanied by acute lung injury (ALI). Ferroptosis plays a role in infectious diseases, but its regulatory mechanism in NS-related ALI remains unclear. The aim of this study is to investigate the mechanism of EMX2OS in promoting ferroptosis in ALI.

**Methods:**

The expression level of EMX2OS in peripheral blood of patients with NS and its diagnostic value were detected by clinical samples. LPS-induced A549 cells were used to establish an ALI model. The targeting relationship between EMX2OS, miR-654-3p and AKT3 was verified by qRT-PCR, CCK-8, detection kit and dual-luciferase assays, and the cell viability and ferroptosis level were evaluated.

**Results:**

EMX2OS was highly expressed in NS and served as a potential diagnostic marker. In LPS-induced lung injury model, high expression of EMX2OS decreased cell viability and enhanced ferroptosis. Silencing EMX2OS had the opposite effects. EMX2OS regulated cell viability and ferroptosis through miR-654-3p/AKT3 axis.

**Conclusions:**

This study reveals for the first time that EMX2OS serves as a diagnostic marker for NS and promotes ferroptosis through miR-654-3p/AKT3 axis, thereby exacerbating lung injury. EMX2OS to regulate ferroptosis may become potential therapeutic strategies for lung injury.

## Introduction

Neonatal sepsis (NS) is a serious infectious disease with high morbidity and mortality, which seriously threatens the life and health of neonates (1). NS is caused by bacteria, fungi and other pathogens invading the blood circulation during the neonatal period, which causes systemic inflammatory response syndrome (SIRS) and leads to multiple organ dysfunction (2). Acute lung injury (ALI) is one of the most common and serious complications of NS (3, 4), which is mainly manifested as alveolar capillary barrier destruction, pulmonary edema and gas exchange dysfunction, which seriously threaten the life health and quality of life of neonates (5, 6). According to the statistics of the World Health Organization, the mortality rate of NS complicated with ALI without timely and effective treatment may exceed 60%, and the survivors may also have sequelae such as chronic lung disease and neurodevelopmental disorders (7, 8). The clinical manifestations of NS are diverse, and early diagnosis is difficult. Therefore, timely diagnosis and treatment are the key to improve the prognosis (9, 10). Traditional diagnostic methods rely on blood culture, C-reactive protein (CRP) and procalcitonin (PCT) detection, but there are limitations such as low sensitivity and long detection cycle (11, 12).

Ferroptosis is an iron-dependent form of non-apoptotic programmed cell death, which is characterized by oxidative stress and cell death caused by intracellular iron accumulation and lipid peroxidation. In recent years, the role of ferroptosis in ALI and acute respiratory distress syndrome (ARDS) has gradually attracted attention, and related research has made important progress (13, 14). *in vitro* studies revealed that after lipopolysaccharide (LPS) intervention, the expression levels of SLC7A11 and GPX4 were significantly downregulated, indicating a weakened antioxidant capacity of the cells. At the same time, the levels of MDA (malondialdehyde) and iron content significantly increased, suggesting an intensified lipid peroxidation reaction and iron metabolism disorder. These changes collectively promoted the occurrence of ferroptosis (13). At present, the specific molecular signaling pathways of ferroptosis in ALI and ARDS are still not fully understood, and the detailed regulatory mechanisms need to be further elucidated (15).

Long non-coding RNA (lncRNA) is a class of RNA molecules that do not encode proteins, and its length is usually more than 200 nucleotides. In ALI studies, lncRNAs were found to be involved in the occurrence and development of the disease through a variety of mechanisms. For example, overexpression of TUG1 significantly ameliorated sepsis-induced ALI, while regulation of lncRNA-ENST contributed to the survival and functional recovery of BMSCs (16, 17). As a lncRNA located in the chromosome 10q24.32 region, EMX2OS has been confirmed to be involved in the regulation of biological processes, such as cell proliferation, apoptosis and oxidative stress (17, 18). At the same time, some studies have shown that EMX2OS is a ferroptosis-related gene and is associated with immune cells (19). In a variety of cancers, EMX2OS acts as an enhancer RNA and may affect cancer progression by regulating gene expression and cell signaling pathways (20). However, its role in NS and associated ALI has not been clearly defined.

The purpose of this study is to analyze the serum expression level of these markers in children, and to evaluate the diagnostic efficacy combined with the receiver operating characteristic (ROC) curve. At the same time, the molecular mechanism of ferroptosis involved in ALI through miR-654-3p/AKT3 pathway was further studied, and the targeted regulatory relationship between key molecules was verified by cell model, so as to provide new ideas and theoretical basis for the prevention and treatment of NS-associated ALI.

## Materials and methods

### Study population

A total of 60 neonates were recruited in Shengli Oilfield Central Hospital from August 2023 to August 2024, including 30 neonates diagnosed with sepsis and 30 healthy neonates as controls. This study was approved by the Research Ethics Committee of Shengli Oilfield Central Hospital (0023427), and written informed consent was obtained from all neonatal guardians. The diagnosis of NS is mainly based on the clinical manifestations, medical history and laboratory examination results of neonates (21). Clinical manifestations meeting three or more of the following symptoms can be determined: (1) hyperthermia or hypothermia; (2) cardiac dysfunction, including tachycardia, brady cardia, and blood pressure reduction; (3) neurological disorders, occurrence of drowsiness, convulsions, epilepsy; (4) apnea, tachypnea, cyanosis; (5) gastrointestinal disorders, such as diarrhea and abdominal distension. Thirty healthy newborns who were born in this hospital during the same period were selected as the control group. Exclusion criteria: (1) premature neonates; (2) congenital malformation or chromosomal abnormality. Blood samples from NS patients and the control group were collected before systematic treatment. Blood samples were centrifuged at 1,200 g for 10 min, and the supernatant was removed and stored at −80 ℃.

### Cell culture and treatment

Human lung epithelial cell line A549 (from the cell Bank of Shanghai Institute of Biochemistry and Cell Biology) was cultured in DMEM/F12 medium containing 10% fetal bovine serum (FBS, AusGeneX, Australia) and 1% double antibody (PWL062, Meilun, China), and cultured in 37 ℃, 5% CO₂ incubator.

LPS (ST1470, Biyuntian, China) 10 mg was prepared into 1 mg/ml mother liquor with sterile water and stored at 4 ℃. When used, the medium was diluted to 10 μg/ml working solution. To investigate the functional role of EMX2OS in ALI, A549 cells were exposed to 10 μg/ml LPS for 24 h.

### Cell transfection

In this study, the small interfering RNA against EMX2OS (si-EMX2OS) and negative controls (scramble) were provided by Santa Cruz Biotechnology, and cell transfection was performed using a Lipofectamine 3000 transfection reagent (Thermo Fisher Scientific). Cells were inoculated into 6-well plates, and cultured to 60% density, and the above vectors were mixed with transfection reagent Lipofectamine 3000 and dropped into the cells. After 6 h, cells were collected for subsequent experiments.

### RNA extraction and quantitative reverse transcription-polymerase chain reaction

RNA samples were extracted from A549 cells and blood using the AG RNAex Pro kit (AG21102, Accurate, China) in accordance with the manufacturer's protocol. The concentration and purity of the RNA were determined using the NanoDrop™ 2000 spectrophotometer (Thermo Scientific™, USA). The OD260/280 ratios for all samples fell within the range of 1.9–2.1. One microgram of total RNA was reverse transcribed into cDNA using the Evo-M-MLV RT Kit with gDNA Clean for qPCR (AG11705, Accurate, China) following the manufacturer's protocol. The RT-PCR was performed using the SYBR Green qPCR Master Mix (Thermo Fisher Scientific, Shanghai, China) by the ABI PRISM 7300 real-time PCR system (Applied Biosystems). The endogenous controls of EMX2OS and AKT3 were GAPDH, and the endogenous controls of miR-654-3p was U6. The thermal cycle was set as follows: 95 ℃ for 1 min and 40 cycles at 95 ℃ for 15 s, 58 ℃ for 20 s and 72 ℃ for 20 s. The final expression values were calculated by the 2^−△△Ct^ method for subsequent analysis.

### Cell viability assay

CCK-8 assay was used to detect the viability of A549 cells in different treatment groups. At different time points after cell transfection, 10 ul CCK-8 reagent (TransGen Biotech, Beijing, China) was added to each well of the 96-well plate, and after continued culture for 2 h, the absorbance value at 450 nm was measured using a microplate reader to calculate cell viability.

### Dual-luciferase reporter assay

Bioinformatics software (ENCORI/starBase available at https://rnasysu.com/encori/) was used to predict the targeted binding relationship between EMX2OS and miR-654-3p, and between miR-654-3p and AKT3. ENCORI/starBase: it is an extended version developed on the basis of starBase, integrating more comprehensive RNA interaction data and analysis functions. It is a comprehensive RNA interaction reference map. The wild-type (Wt) and mutant (Mut) miR-654-3p binding site sequences in the EMX2OS and AKT3 3′-UTR were integrated into the pmirGLO vector for the generation of the Wt vector and Mut vector. miR-654-3p mimic or inhibitor was co-transfected with EMX2OS-Wt or EMX2OS-Mut and AKT3-Wt or AKT3-Mut into cells with Lipofectamine 3000. After 48 h, cells were harvested using the lysate from a commercial kit and relative luciferase activity was measured.

### Iron assay

According to the Ferrozine colorimetric assay kit (TC0983, Leagene), the cell homogenate supernatants were used to measure iron level. Next, the cells were centrifuged at 13,000 g for 10 min at 4 °C to obtain the supernatant. In a centrifuge tube, the supernatant was incubated with buffer for 10 min at 37 °C. The OD was determined at 562 nm. The sample size for each experimental group was *n* = 6. Additionally, each group of experiments was independently repeated three times.

### Detection of lipid peroxides

The content of lipid oxidation in cells was detected by MDA detection kit (S0131S, Beyotime) following the manufacturer's instructions. After the cells were broken by ultrasound, the supernatant was collected, and MDA detection reagents were added followed by 30 min incubation at 90 ℃. The absorbance of the supernatant at 532 nm and 600 nm was detected, and MDA levels were calculated. The sample size for each experimental group was *n* = 6. Additionally, each group of experiments was independently repeated three times.

### Glutathione examination

GSH (glutathione) assay kit (S0053, Beyotime) was utilized to measure the level of GSH. After the cells were broken by ultrasound, the supernatant was collected, and GSH detection reagents were added. The GSH concentration was obtained by measuring absorbance at 412 nm.

### Analysis of the diagnostic efficacy of EMX2OS as a diagnostic marker for NS

The diagnostic value of EMX2OS for NS was evaluated using the ROC curve analysis: ROC curve was potted based on the detection value of EMX2OS, and the diagnosis results of NS were assigned values for statistical analysis (GraphPad Prism, version 9.5.1) (1 for having the disease, 0 for not having the disease). The area under the ROC curve (AUC) was calculated. The closer the AUC value is to 1, the better the diagnostic efficacy of EMX2OS is (AUC > 0.7 indicates certain diagnostic value, AUC > 0.8 indicates good diagnostic value, and AUC > 0.9 indicates excellent diagnostic value). Determine the optimal cut-off value: By calculating (sensitivity + specificity), select the EMX2OS detection value corresponding to the maximum Youden index as the optimal cut-off value. Based on the optimal critical value, the sensitivity and specificity of EMX2OS in diagnosing NS were calculated respectively to comprehensively evaluate its diagnostic efficacy.

### Statistical analysis

All assays were conducted in triplicates, using GraphPad Prism 9.0 and SPSS 27.0 for statistical analysis. All the measurement data in this study were normally distributed. Data were presented as the mean ± SD. The statistical tests of two-tailed student's t-test or one-way analysis of variance (ANOVA) followed by Tukey test were employed to ascertain the significant differences between the groups. ROC curve was drawn to evaluate the diagnostic value of EMX2OS for NS. Each group of experiments was independently repeated three times. *P* < 0.05 indicates that the difference is statistically significant.

## Result

### EMX2OS is a diagnostic marker for NS

**Figure 1:**
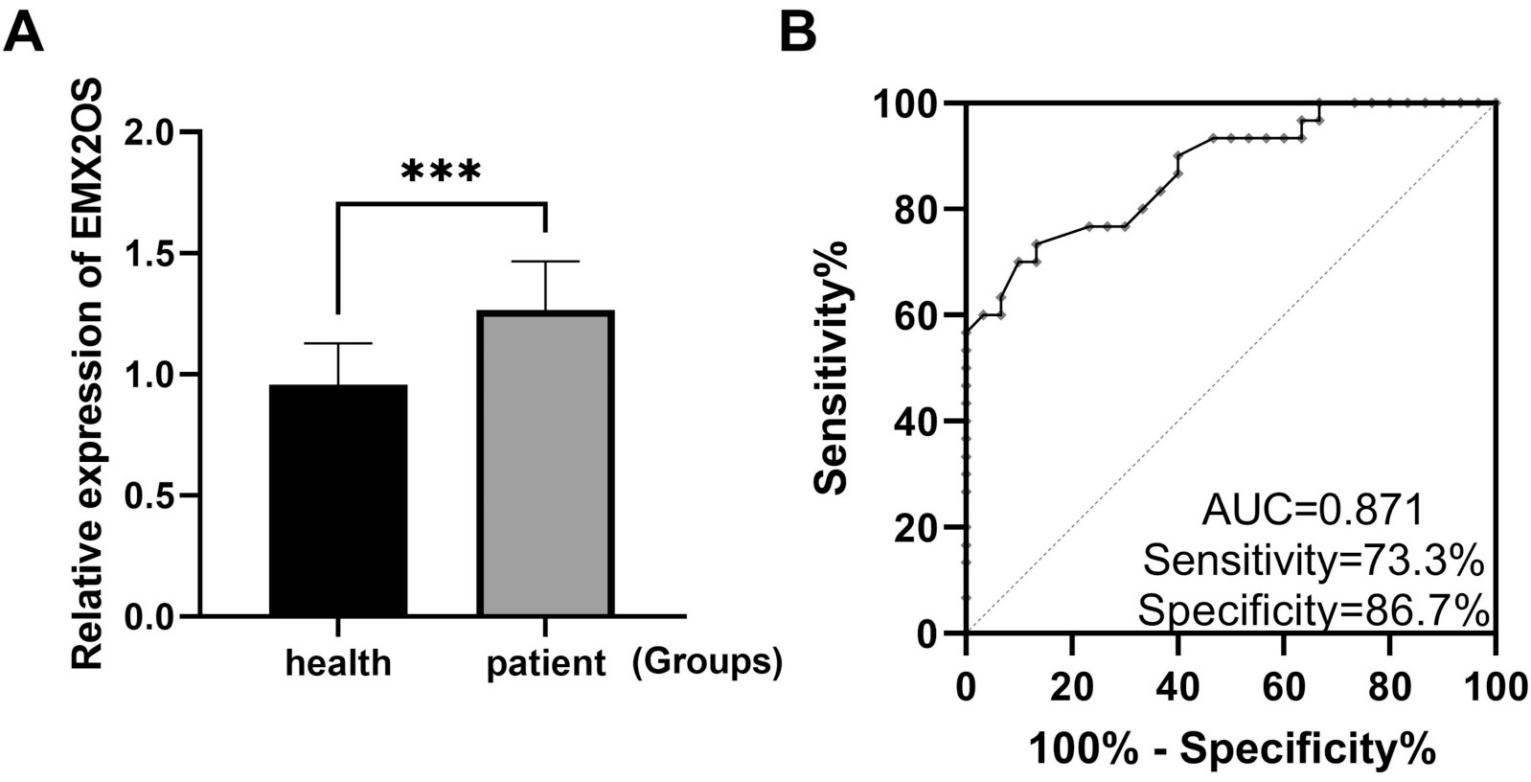
EMX2OS is a diagnostic marker for NS. **(A)** RT-qPCR confirmed that the expression level of EMX2OS in the serum of NS was significantly higher than that of healthy neonates (***P *<* 0.001). **(B)** ROC curves plotted based on EMX2OS in serum.

The expression level of EMX2OS in the serum of neonates with sepsis was significantly higher than that of healthy neonates (Figure 1A, *P* < 0.001). The ROC curve was drawn to evaluate the diagnostic value of EMX2OS for NS (Figure 1B), and the results showed that the area under the ROC curve (AUC) was 0.871, the sensitivity was 73.3%, and the specificity was 86.7%.

### Cell viability and ferroptosis in LPS-constructed lung injury model

**Figure 2:**
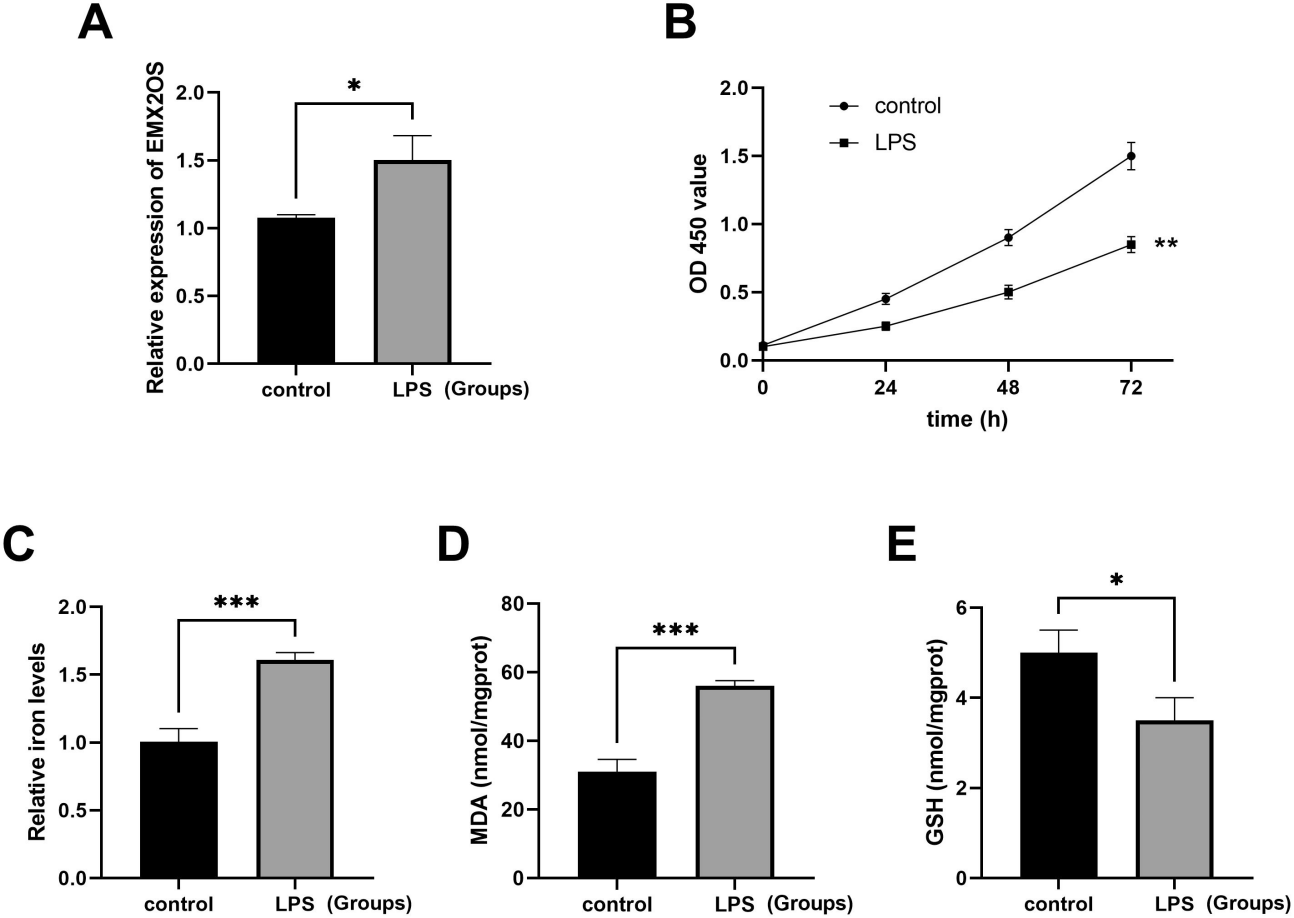
The ALI model was induced by LPS. **(A)** The expression level of the EMX2OS gene was detected by qRT-PCR to reflect the effect of LPS stimulation on the expression of EMX2OS. EMX2OS was highly expressed after LPS treatment (*P < 0.05). **(B)** The cell proliferation was detected through the CCK-8 assay, which showed the differences in cell proliferation between the LPS-stimulated group and the control group at different time points (0, 24, 48, and 72 h). Cell viability was significantly reduced after LPS treatment (** *P* < 0.01). **(C)** The “Relative iron levels” on the *y*-axis refers to the relative iron content. Through the use of the test kit, the changes in intracellular iron levels after LPS stimulation are shown (*** *P* < 0.001). **(D,E)** MDA (malondialdehyde) and GSH (glutathione) concentrations in the cells were detected by kits. Reflects the degree of lipid peroxidation and the cell's antioxidant capacity (* *P* < 0.05, *** *P* < 0.001). Data represent three independent experiments. Statistical analysis was performed using an independent samples t-test.

The ALI model was established by LPS treatment, and the expression of EMX2OS was significantly higher than that in the control group (Figure 2A, *P* < 0.05). The viability of A549 cells in the LPS model group was significantly decreased (Figure 2B, *P* < 0.01), while the iron ion content was increased (Figure 2C, *P* < 0.001), the MDA level was significantly increased (Figure 2D, *P* < 0.001), but the GSH level was decreased (Figure 2E, *P* < 0.05).

### EMX2OS acts as a molecular sponge for miR-654-3p

**Figure 3:**
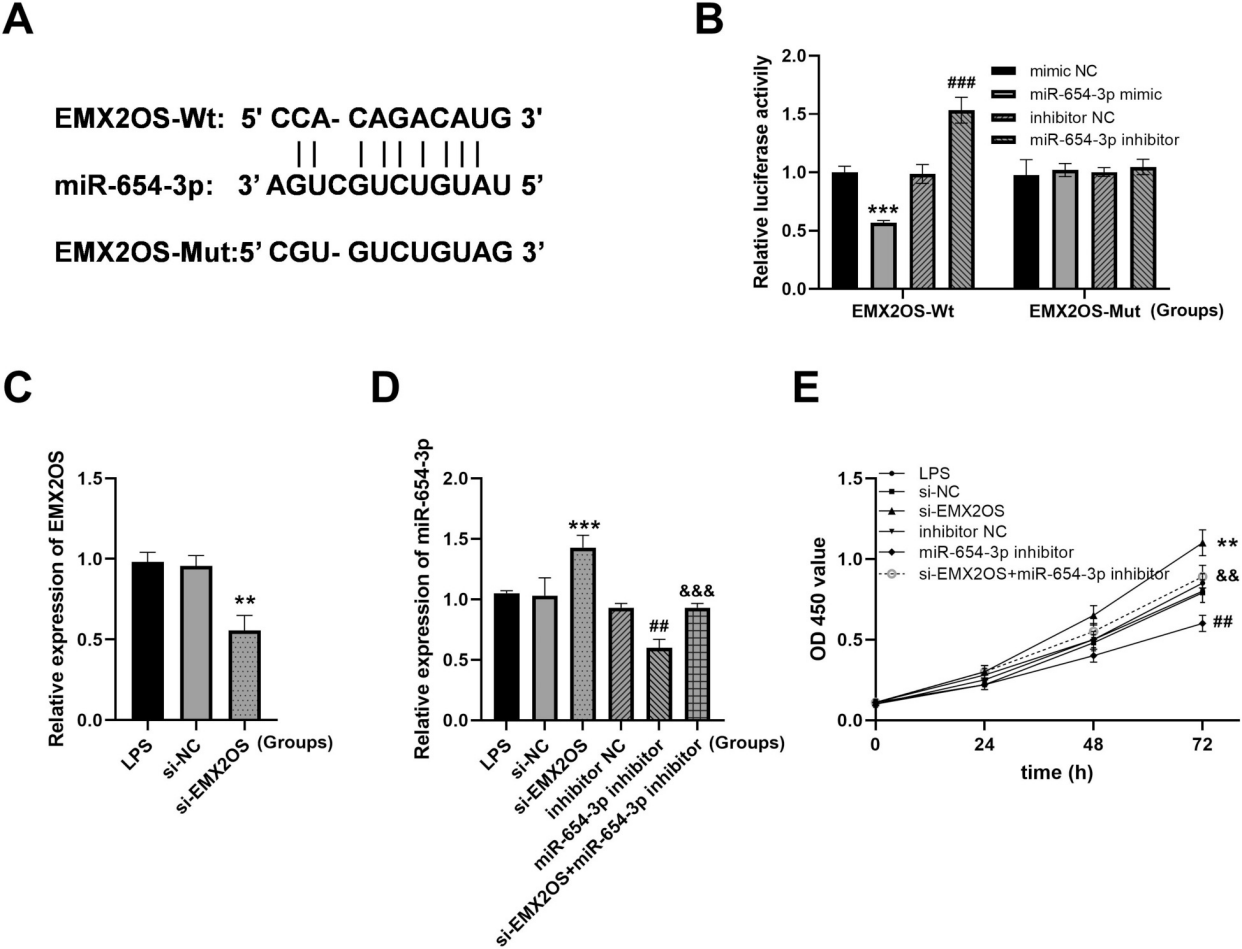
EMX2OS acts as a molecular sponge for miR-654-3p. **(A)** Bioinformatics database StarBase identified the presence of EMX2OS binding sequence to miR-654-3p. **(B)** Dual-luciferase reporter assay to authenticate the targeted between miR-654-3p and EMX2OS in A549 (*** *P* < 0.001 vs. mimic NC; ### *P* < 0.001 vs. inhibitor NC). Data were presented as the mean ± SD (*n* = 3). **(C)** Expression of EMX2OS was decreased after transfection with si-EMX2OS (** *P* < 0.01 vs. si-NC). **(D)** RT-qPCR of miR-654-3p in cell model after con-transfection with si-EMX2OS and miR-654-3p inhibitor (*** *P* < 0.001 vs. si-NC; ## *P* < 0.01 vs. inhibitor NC; &&& *P* < 0.001 vs. si-EMX2OS). **(E)** Cell viability was detected by CCK-8 assay (** *P* < 0.01 vs. si-NC; ## *P* < 0.01 vs. inhibitor NC; && *P* < 0.01 vs. si-EMX2OS). Data were presented as the mean ± SD (*n* = 3).

We identified the binding site of EMX2OS to miR-654-3p through a bioinformatics database (starBase) (Figure 3A). The results of the dual-luciferase reporter assay showed (Figure 3B) that after transfection with miR-654-3p mimic, the luciferase activity of the EMX2OS-Wt reporter gene significantly decreased (*P* < 0.001); while after transfection with miR-654-3p inhibitor, the luciferase activity of EMX2OS-Wt significantly increased (*P* < 0.001). Regardless of whether miR-654-3p mimic or inhibitor was used for transfection, the luciferase activity of the EMX2OS-Mut reporter gene did not show any statistical difference compared with the respective negative controls (mimic NC and inhibitor NC) (*P* > 0.05).

As shown in Figure 3C, the expression of EMX2OS was significantly downregulated in cells transfected with si-EMX2OS (*P* < 0.01). si-EMX2OS group: the expression of miR-654-3p was significantly upregulated (Figure 3D, *P* < 0.001), suggesting that the knockdown of EMX2OS might relieve the inhibitory effect on miR-654-3p. miR-654-3p inhibitor group: the expression of miR-654-3p was expectedly downregulated (Figure 3D, *P* < 0.01), verifying the effectiveness of the inhibitor. si-EMX2OS+miR-654-3p inhibitor group: compared with the single knockdown of EMX2OS, the combined inhibitor treatment could reverse the high expression of miR-654-3p induced by si-EMX2OS (Figure 3D, *P* < 0.001). The si-EMX2OS treatment significantly enhanced the cell proliferation ability (Figure 3E, *P* < 0.01), while the miR-654-3p inhibitor alone inhibited proliferation (Figure 3E, *P* < 0.01). After combining si-EMX2OS with the miR-654-3p inhibitor, the proliferation ability partially recovered (Figure 3E, *P* < 0.01).

### Effect of EMX2OS on ferroptosis via miR-654-3p

**Figure 4:**
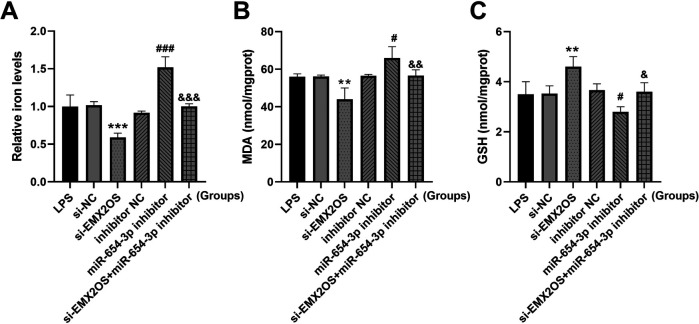
miR-654-3p mediates the regulation of ferroptosis by EMX2OS. **(A)** Iron levels in the cells were detected by iron assay kit. **(B,C)** MDA and GSH concentrations in the cells were detected by kits. Data were presented as the mean ± SD (*n* = 3). Statistical analysis was performed using one-way ANOVA followed by Tukey's multiple comparisons test. ** *P* < 0.01, *** *P* < 0.001 vs. si-NC; # *P* < 0.05, ### *P* < 0.001 vs. inhibitor NC; & *P* < 0.05, && *P* < 0.01, &&& *P* < 0.001 vs. si-EMX2OS.

Compared with the si-NC group, the intracellular iron content and MDA level in the si-EMX2OS group were significantly decreased (Figures 4A,B, *P* < 0.01), and the GSH content was increased (Figure 4C, *P* < 0.05). However, miR-654-3p inhibitor group had significantly increased iron content and MDA level (Figures 4A,B, *P* < 0.05), and decreased GSH content (Figure 4C, *P* < 0.05). Notably, the levels of iron and MDA in the si-EMX2OS+miR-654-3p inhibitor group were significantly higher than those in the si-EMX2OS group, and the GSH content was significantly lower (Figures 4A–C, *P* < 0.05).

### EMX2OS/miR-654-3p/AKT3 regulated the viability of ALI model cells

**Figure 5:**
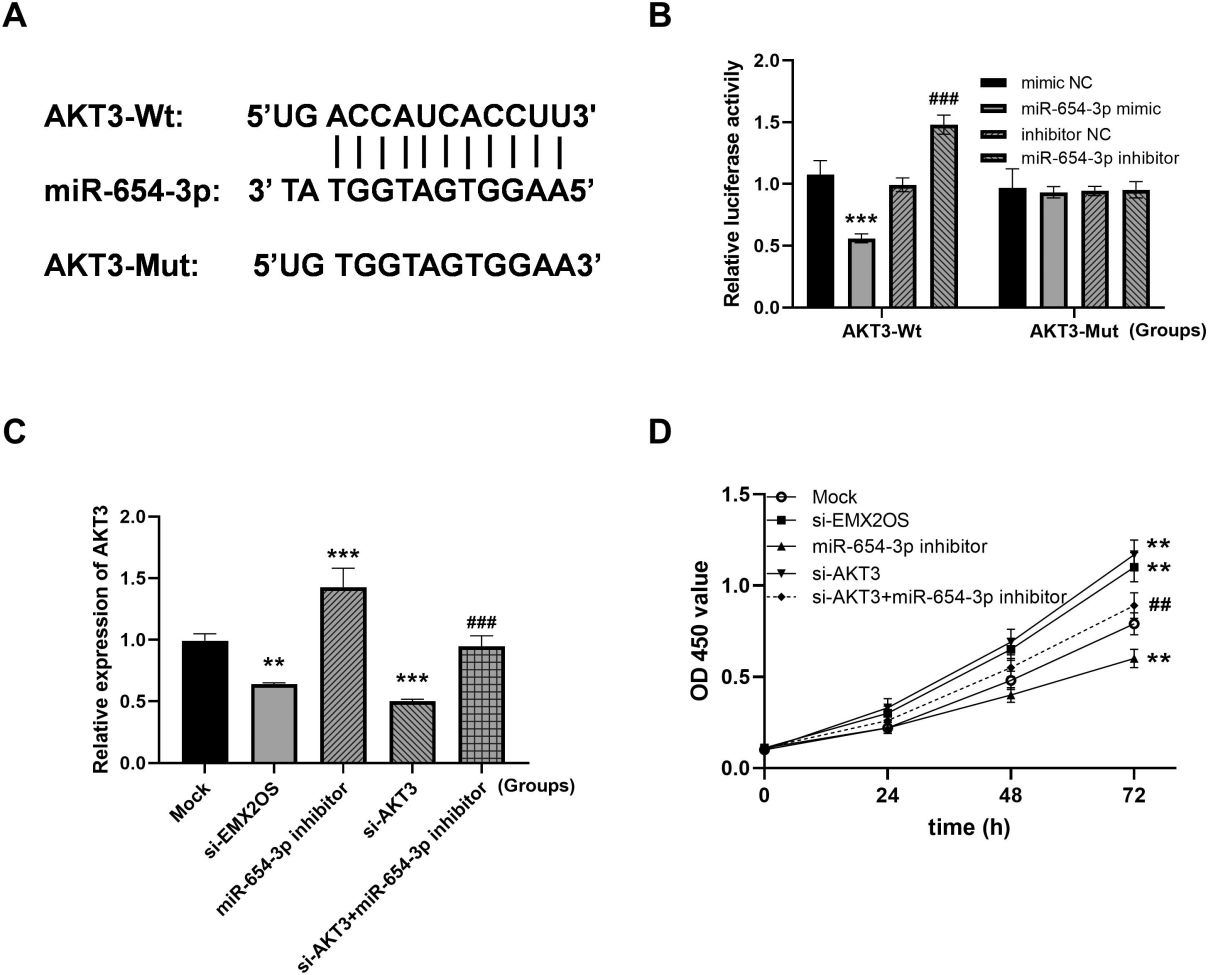
EMX2OS regulates cell viability through miR-654-3p/AKT3 (AKT serine/threonine kinase 3). **(A)** The binding site of miR-654-3p to AKT3. **(B)** Dual-luciferase reporter assay to authenticate the targeted between miR-654-3p and AKT3 in A549 (*** *P* < 0.001 vs. mimic NC; ### *P* < 0.001 vs. inhibitor NC). Data were presented as the mean ± SD (*n* = 3). **(C)** The expression level of AKT3. **(D)** Cell viability was examined in co-transfected cells. (** *P* < 0.01, *** *P* < 0.001 vs. Mock; ## *P* < 0.01, ### *P* < 0.001 vs. miR-654-3p inhibitor). Data were presented as the mean ± SD (*n* = 3).

Next, we identified a potential interaction between miR-654-3p and AKT3 (Figure 5A). miR-654-3p mimics inhibited and miR-654-3p inhibitors increased the luciferase activity of AKT3 3’UTR (Figure 5B, both *P* < 0.001). In addition, AKT3 expression was significantly promoted by miR-654-3p inhibitor, but inhibited by reduced EMX2OS, and its expression was successfully decreased by si-AKT3 (Figure 5C, all *P* < 0.01).

Compared with the Mock group, the viability of ALI model cells in the si-EMX2OS group and the si-AKT3 group were significantly increased (Figure 5D, *P* < 0.01). However, the cell viability in the miR-654-3p inhibitor group was significantly decreased (Figure 5D, *P* < 0.01). Notably, the cell viability of the si-AKT3+miR-654-3p inhibitor group was significantly higher than that of the miR-654-3p inhibitor group (Figure 5D, *P* < 0.01).

### EMX2OS regulates ferroptosis through miR-654-3p/AKT3

**Figure 6:**
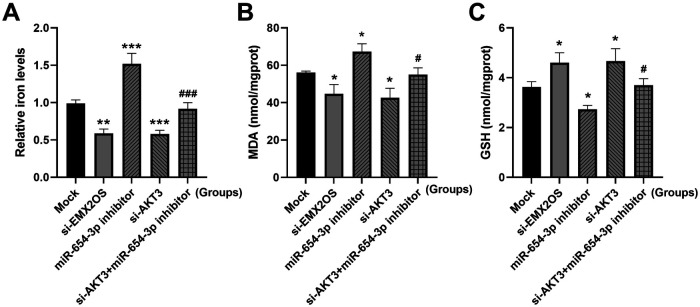
EMX2OS regulates ferroptosis through miR-654-3p/AKT3. **(A)** Iron levels in the cells were detected by iron assay kit. **(B,C)** MDA and GSH concentrations in the cells were detected by kits. Data were presented as the mean ± SD (*n* = 3). Statistical analysis was performed using one-way ANOVA followed by Tukey's multiple comparisons test. * *P* < 0.05, ** *P* < 0.01, *** *P* < 0.001 vs. Mock; # *P* < 0.05, ### *P* < 0.001 vs. miR-654-3p inhibitor.

Compared with the Mock group, the intracellular iron content and MDA level were significantly decreased, and the GSH content was significantly increased (Figures 6A–C, *P* < 0.05) after EMX2OS and AKT3 knockdown. However, the miR-654-3p inhibitor treatment group showed the opposite trend, with increased iron and MDA levels and decreased GSH content (Figure 6A, *P* < 0.05). In addition, co-transfection of si-AKT3 significantly reversed the promoting effect of miR-654-3p inhibitor on ferroptosis, as shown by the decrease of iron and MDA levels and the increase of GSH content (Figure 6A, *P* < 0.05).

## Discussion

NS is the most serious systemic infectious disease in the neonatal period. The pathogenesis of NS mainly involves bacteria, viruses and other pathogens breaking through the body's immune barrier and spreading through the blood circulation system, leading to multiple organ dysfunction syndrome (MODS) (2). ALI is one of the most common complications of NS, and its pathological process involves complex inflammatory cascades and cell metabolic disorders, which seriously threaten the life and health of children and long-term quality of life (3). At present, the early diagnosis and effective treatment of NS complicated with ALI are still the key scientific problems to be solved in the field of perinatal medicine. This study found that lncRNA EMX2OS was significantly up-regulated in peripheral blood of patients with NS and had high diagnostic value. Mechanistic studies confirmed that EMX2OS promoted ferroptosis through miR-654-3p/AKT3 pathway, thereby promoting the development of ALI pathological process.

EMX2OS has shown significant diagnostic and prognostic value in a variety of cancers, such as gastric cancer and renal clear cell carcinoma, where its expression level is significantly correlated with patient grade, stage and cancer status (22, 23). In the present study, the expression level of EMX2OS was found to be significantly higher in samples from NS patients than in healthy controls by qRT-PCR. Then the ROC curve was drawn to evaluate the efficacy of EMX2OS in the diagnosis of NS. The results showed that EMX2OS had a high AUC, and EMX2OS had good sensitivity and specificity in NS. It is expected to be used in the clinical auxiliary diagnosis of NS, which is helpful to early detection of the disease, timely intervention and treatment, and improve the prognosis of children.

This study selected the A549 cell line for the experiments, based on the following considerations of practical feasibility and research requirements: the accessibility and stability of the research model. The acquisition of primary lung epithelial cells from newborns is extremely difficult due to ethical restrictions. On one hand, samples of newborn lung tissue (especially normal lung tissue) are extremely hard to obtain due to ethical limitations. On the other hand, the primary cell isolation and culture techniques are complex, and there are significant individual differences, making it difficult to ensure the reproducibility of the experiments. However, the A549 cell line, as an internationally recognized research model related to lung epithelium, has a stable source, is easy to culture, and has strong proliferation ability. It can complete a large number of repeated experiments under standardized conditions, ensuring the stability of the study on the iron death-related mechanism regulated by EMX2OS. In the ALI cell model, EMX2OS was significantly highly expressed, the cell viability was low, and the ferroptosis was promoted. Knockdown of EMX2OS could effectively alleviate this process. The results of the present study suggest that EMX2OS may aggravate acute lung injury by promoting ferroptosis. This result echoes the conclusion that ferroptosis is involved in the pathological process of lung diseases in many studies. For example, ferroptosis has been shown to aggravate lung tissue injury in acute respiratory distress syndrome (24). This study further clarified the role of EMX2OS in the regulation of cell fate in the pathological development of ALI, and complemented the mechanistic studies of lncRNAs affecting cell viability and ferroptosis in lung diseases.

The dual-luciferase reporter assay confirmed the targeted binding relationship between EMX2OS and miR-654-3p, and miR-654-3p and AKT3. miR-654-3p partially reversed the effects of low expression of EMX2OS on cell viability and ferroptosis, and low expression of AKT3 partially reversed the effects of miR-654-3p on cell viability and ferroptosis. This study confirmed that EMX2OS regulated AKT3 expression through sponge adsorption of miR-654-3p, thereby promoting ferroptosis. Unlike the pro-survival role of the PI3K/AKT pathway in cell survival reported in other studies (25), this study is a breakthrough to reveal a novel function of AKT3 in the regulation of ferroptosis. Traditionally, AKT, as the core protein of the PI3K/AKT pathway, inhibits apoptosis and maintains cell survival by phosphorylating downstream target proteins. However, in the pathological context of NS with ALI, the present study found that AKT3 promoted iron and MDA, thereby accelerating the process of ferroptosis. AKT3 may also act on the key molecules of ferroptosis through a pathway independent of PI3K. Previous studies have shown that AKT3 can regulate the activity and stability of ACSL4 by phosphorylating it (26). ACSL4 is a key driver of ferroptosis, promoting lipid peroxidation and providing a material basis for ferroptosis (27). AKT3 phosphorylates the Ser26 site of SLC7A11, inhibiting its cystine transport activity and reducing GSH levels (28). SLC7A11/SLC3A2 mediates cystine uptake, which is the rate-limiting step in GSH synthesi (29). This finding improves the mechanism of AKT3 in the pathological process of diseases and provides a solid theoretical basis for exploring interventions targeting this pathway. Based on this, future studies can develop small molecule inhibitors or RNA interference technology specifically targeting EMX2OS/miR-654-3p/AKT3 axis, which may provide a new treatment strategy for improving the prognosis of neonatal sepsis.

Although this study has achieved some results, it still has limitations. On the one hand, the study samples were mainly from a single region, and the sample size was limited, which may lead to bias caused by regional and individual differences. On the other hand, only cell-level studies have been conducted and animal model validation is lacking. The selection of cell models is also an important limitation of this study. The A549 cells originate from adult lung adenocarcinoma cells, and there are significant differences in their genetic background, proliferation characteristics, and signal pathway regulation compared to normal neonatal lung epithelial cells (especially immature type II alveolar epithelial cells). Such common chromosomal abnormalities and metabolic pathway disorders in cancer cell lines may lead to deviations in their response to ferroptosis and the regulation mode of EMX2OS from that of normal cells, making it impossible to fully simulate the physiological state of neonatal lung epithelium. At the same time, we have listed “using primary neonatal lung epithelial cells or induced pluripotent stem cells (iPSC) differentiated alveolar epithelial cells for verification” as the next research focus, in order to deeply explore the role of EMX2OS with a model that is closer to the physiological state. In the subsequent studies, we will prioritize the establishment of an LPS-induced ALI model in newborn mice. By detecting the expression of EMX2OS in lung tissue, iron death-related indicators (such as lipid peroxidation levels) and lung injury pathological scores, we will further verify the role of EMX2OS in the *in vivo* environment. Meanwhile, future studies can expand the sample scope, carry out multi-center clinical research, in-depth explore the EMX2OS-related regulatory network, and explore targeted intervention strategies, so as to open up a new path for the prevention and treatment of neonatal sepsis and related complications.

In summary, the present study confirmed that EMX2OS can be used as a potential biomarker for the diagnosis of NS and is involved in the pathological process of NS-associated ALI by promoting ferroptosis through miR-654-3p/AKT3 signaling pathway. This finding not only enriches the understanding of the pathogenesis of NS complicated with ALI, but also provides a new target and theoretical basis for early diagnosis and targeted therapy of the disease.

## Data Availability

The original contributions presented in the study are included in the article/Supplementary Material, further inquiries can be directed to the corresponding author.
